# Ferroptosis in Intrahepatic Cholangiocarcinoma: *IDH1^105GGT^* Single Nucleotide Polymorphism Is Associated With Its Activation and Better Prognosis

**DOI:** 10.3389/fmed.2022.886229

**Published:** 2022-07-08

**Authors:** Samantha Sarcognato, Diana Sacchi, Luca Fabris, Giacomo Zanus, Enrico Gringeri, Monia Niero, Giovanna Gallina, Maria Guido

**Affiliations:** ^1^Department of Pathology, Azienda ULSS2 Marca Trevigiana, Treviso, Italy; ^2^Department of Molecular Medicine – DMM, University of Padova, Padova, Italy; ^3^4th Surgery Unit, Azienda ULSS2 Marca Trevigiana, Treviso, Italy; ^4^Department of Surgery, Oncology and Gastroenterology – DISCOG, University of Padova, Padova, Italy; ^5^Department of Medicine – DIMED, University of Padova, Padova, Italy

**Keywords:** intrahepatic cholangiocarcinoma, ferroptosis, *IDH1*^105*GGT*^ single nucleotide polymorphism, STAT3, GPX4

## Abstract

**Objectives:**

Intrahepatic cholangiocarcinoma (ICC) has a dismal prognosis and often demonstrates an anti-apoptotic landscape, which is a key step to chemotherapy resistance. Isocitrate dehydrogenase 1 or 2 (*IDH1-2*)-mutated ICCs have been described and associated with better prognosis. Ferroptosis is a regulated iron-mediated cell death induced by glutathione peroxidase 4 (GPX4) inhibition, and may be triggered pharmacologically. GPX4 is overexpressed in aggressive cancers, while its expression is inhibited by *IDH1*^*R*132*C*^ mutation in cell lines. We investigated tissue expression of ferroptosis activation markers in ICC and its correlation with clinical-pathological features and *IDH1-2* status.

**Materials and Methods:**

We enrolled 112 patients who underwent hepatic resection or diagnostic liver biopsy for ICC. Immunostaining for transferrin-receptor 1 and GPX4, and Pearls’ stain for iron deposits were performed to evaluate ferroptosis activation. Immunostaining for STAT3 was performed to study pro-inflammatory and anti-apoptotic landscape. Main *IDH1-2* mutations were investigated in 90 cases by real-time polymerase chain reaction.

**Results:**

GPX4 overexpression was seen in 79.5% of cases and related to poor histological prognostic factors (grading and perineural and vascular invasion; *p* < 0.005 for all) and worse prognosis (OS *p* = 0.03; DFS *p* = 0.01). STAT3 was expressed in 95.5% of cases, confirming the inflammation-related anti-apoptotic milieu in ICC, and directly related to GPX4 expression (*p* < 0.0001). A high STAT3 expression correlated to a worse prognosis (OS *p* = 0.02; DFS *p* = 0.001). Nearly 12% of cases showed *IDH1*^105*GGT*^ single nucleotide polymorphism, which was never described in ICC up to now, and was related to lower tumor grade (*p* < 0.0001), longer overall survival (*p* = 0.04), and lower GPX4 levels (*p* = 0.001).

**Conclusion:**

Our study demonstrates for the first time that in most inflammatory ICCs ferroptosis is not active, and its triggering is related to *IDH1-2* status. This supports the possible therapeutic role of ferroptosis-inducer drugs in ICC patients, especially in drug-resistant cases.

## Introduction

Intrahepatic cholangiocarcinoma (ICC) is the second most common primary liver tumor, whose incidence and mortality have increased worldwide over the last decades (1–6). Because of a frequent diagnosis at an advanced stage, ICC prognosis remains dismal. Surgical resection is the only potentially curative treatment option, but recurrence rates remain high. Patients with metastatic or unresectable disease undergo palliative non-curative systemic therapies, with only modest increases in overall survival and frequent development of chemoresistance, often due to an escape from drug-induced apoptosis by cancer cells (1, 3–5, 7, 8).

Advances have been made in the last decade regarding ICC molecular background. In particular, Sia et al. identified two molecular subclasses, named the proliferative and the inflammation-related classes (9). The latter is defined by the triggering of pro-inflammatory signaling pathways *via* different interleukins and the signal transducer and activator of transcription 3 (STAT3) protein (4, 9). STAT3 is involved in several cellular processes, inhibits apoptosis, and it is known to play a role in many cancer types, being associated with a worse prognosis (4, 10–13).

A subgroup of ICCs showing missense mutations in the isocitrate dehydrogenase 1 and 2 (*IDH1-2*) genes has also been described (14, 15). *IDH*-mutated ICCs exhibit high expression of mitochondrial genes and low expression of chromatin modifier genes and were demonstrated to have a better prognosis than cases with wild type *IDH1-2* (3, 4, 14–17).

Ferroptosis is a newly described form of regulated iron-mediated cell death type, whose activation requires high intracytoplasmic iron concentrations and the inhibition of the reduced glutathione (GSH)-dependent enzyme glutathione peroxidase 4 (GPX4) (18, 19). GPX4 overexpression has been described in many aggressive cancers, making it a potential therapeutic target able to promote death in drug-tolerant tumor cells. Many molecules currently exist that are able to trigger the ferroptotic cascade, known as ferroptosis inducers, either by decreasing GSH levels, such as erastin, or by directly inhibiting GPX4 activity, such as RSL3. Some of them are currently under investigation in clinical trials for cancer treatment (19, 20). So far, no data are reported in the literature regarding the possible role of ferroptosis and ferroptosis inducers in ICC.

An *in vitro* study by Wang et al. (21) conducted on different cell lines firstly demonstrated that tumor-derived *IDH1*^*R*132*C*^ mutation sensitizes cells to ferroptosis, by reducing GPX4 levels through the production of the oncometabolite 2-hydroxyglutarate (2-HG). In their work, mutated cells were able to undergo ferroptosis in response to erastin but not to RSL3, which acts in a concentration-dependent manner, implying that 2-HG acts directly on GPX4 expression (21).

Basing on this background, the aim of our study was to investigate tissue expression of ferroptosis activation markers in ICC cases, and to correlate it with clinical-pathological features, STAT3 expression, and *IDH1-2* status.

## Materials and Methods

### Case Selection

We retrospectively collected a total of 112 consecutive patients with a diagnosis of ICC. Among them, 90 patients underwent laparoscopic hepatic resection with curative intent from January 2006 to May 2021. The remaining 22 patients were patients with liver mass who underwent diagnostic liver biopsy, eventually diagnosed as ICC, who subsequently underwent surgery in a different hospital. Thirty patients included in our cohort were already investigated before, as part of a previous study (22). Exclusion criteria were (i) the administration of any systemic or loco-regional therapy prior to surgery/biopsy, (ii) a survival of less than 3 months after surgery (for patients who underwent resection), to exclude deaths due to surgical complications, and (iii) the absence of available residual tumor tissue for immunohistochemical (IHC) stains and molecular tests. Only surgical specimens were considered for molecular analyzes. The study complies with the ethical guidelines of the 1975 Declaration of Helsinki and obtained the approval from the local Ethics Committee (Ethics Committee for Clinical Research—University Hospital of Padova, Italy; protocol #: 0038038/17). All of the patients gave their appropriate informed consent to any procedure.

### Clinical Data

Patients’ relevant clinical and laboratory data were retrieved from medical records, including sex, age, serum ferritin levels, the presence of any underlying chronic hepatic or biliary disease, the presence of cirrhosis, and the administration of adjuvant chemotherapy. We also reported whether lymphadenectomy was performed during surgery or not.

All patients who underwent surgery were clinically followed-up, and regularly subjected to ultrasonography and computed tomography to detect any recurrence of the disease. Overall and disease-free survival time was obtained from medical charts.

### Histological Study

All of the cases were blindly and contemporarily reviewed by an experienced (MG) and two trainee (SS and DS) liver pathologists, and relevant histological features were recorded, including macroscopic tumor type, histotype, grade of differentiation, T stage (according to the revised 8th edition of the UICC staging system) (23), margin status (for surgical resections), and the presence of vascular and perineural invasion and lymph node metastasis.

### Immunohistochemical Study

Tissue microarrays (TMAs) made of formalin-fixed paraffin-embedded ICC tissue cores (with a diameter of 2 mm) were obtained by selecting two or three representative tumor areas from each liver resection case, depending on tumor dimension. All of the samples were processed by using the TMA Master platform (3DHistech, Budapest, Hungary), a semi-automatic and computer-assisted TMA platform.

**FIGURE 1:**
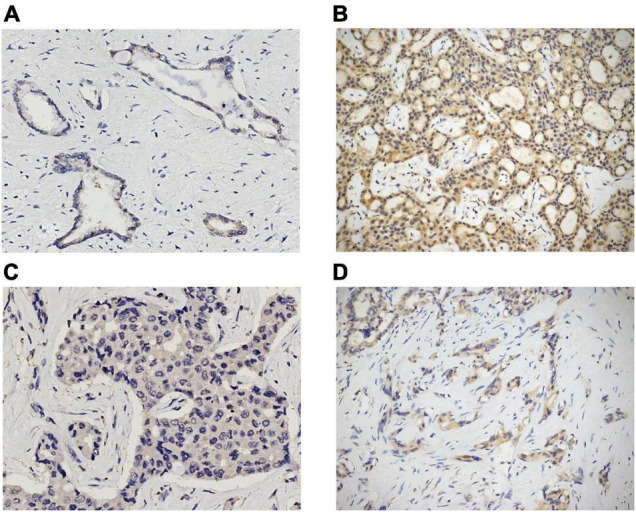
STAT3 and GPX4 expression in ICC cases. Examples of 1 + and 2 + cytoplasmic positivity for STAT3 and GPX4 [**(A)** 1 + STAT3, original magnification 20x; **(B)** 2 + STAT3, original magnification 10x; **(C)** 1 + GPX4, original magnification 20x; **(D)** 2 + GPX4, original magnification 10x].

**FIGURE 2:**
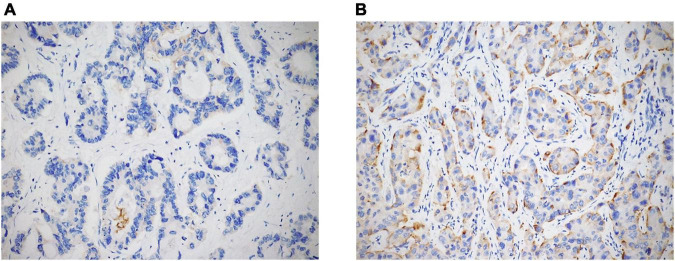
TFR1 expression in ICC cases was semi-quantitatively evaluated as negative, 1 + [**(A)** original magnification 20x], and 2 + [**(B)** original magnification 20x].

Immunostains were performed on TMA and liver biopsy sections by using the following antibodies: anti-STAT3 (clone F-2; Santa Cruz Biotechnology, Dallas, TX, United States; dilution 1:200; mouse monoclonal), anti-GPX4 (clone E-12; Santa Cruz Biotechnology, Dallas, TX, United States; dilution 1:400; mouse monoclonal), and anti-transferrin receptor 1 (TFR1; also known as CD71) (clone 10F11; Leica Biosystems, Newcastle upon Tyne, United Kingdom; dilution 1:100; mouse monoclonal). All IHC stains were conducted according to standard protocols by using the Dako Omnis autostainer (Dako, Glostrup, Denmark), and all of the slides were counterstained with hematoxylin. Appropriate positive and negative controls were used for each run. In evaluating the expression of all markers, only cytoplasmic staining was considered. STAT3 and GPX4 expression was semi-quantitatively scored from 0 to 2 +, as follows: 0 = negative; 1 + = expression in ≤ 50% of tumor cells (TCs); 2 + = expression in > 50% of TCs (Figure 1). Finally, TFR1 positivity was evaluated as follows: 0 = negative or positive in ≤ 10% of TCs; 1 + = expression in 11–50% of TCs; 2 + = expression in > 50% of TCs (Figure 2).

**FIGURE 3:**
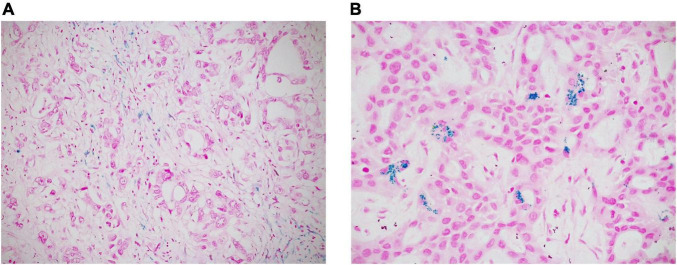
Intratumoral iron deposits in ICC were evaluated as absent/present. Negative cases often showed iron deposition in peri-tumoral macrophages, but not in neoplastic cells [**(A)** original magnification 20x]; in positive cases, iron deposits appear as intracytoplasmic blue granules in tumor cells [**(B)** original magnification 40x].

In every case, we also performed histochemical Perls’ stain (Artisan Iron Stain Kit, Dako, Glostrup, Denmark) to detect intratumoral iron deposits, which were recorded as absent/present (Figure 3).

### Molecular Study

As reported above, only surgical specimens (90 cases) were considered for molecular tests. For each case, DNA extraction was performed on selected representative areas of formalin-fixed paraffin-embedded ICC tissue, with Qiasymphony DSP DNA Mini Kit (Qiagen, Hilden, Germany). DNA quantity was measured spectrophotometrically by using NanoDrop ND 100 Spectrophotometer (Thermo Fisher Scientific, Waltham, MA, United States). Detection of the main *IDH1* (codons 105 and 132) and *IDH2* (codons 140 and 172) gene mutations was performed by real-time polymerase chain reaction (PCR) analysis (Easy PGX ready IDH1-2, Easy PGX platform, Diatech Pharmacogenetics, Jesi, Italy), according to the manufacturer’s instructions. When necessary, the identified mutations were confirmed by PCR analysis and Sanger sequencing, by using the ABI PRISM 3500 Genetic Analyzer (Applied Biosystems, Waltham, MA, United States).

### Statistical Analysis

Continuous variables were expressed as median (range) while categorical variables as frequency and percentage. For clinical-pathological correlations, appropriated tests were used, including Student’s *t*-test, one-way ANOVA test, Spearman rank correlation test and Fisher exact probability test. The Kaplan–Meier method was used to create survival curves, which were compared by using both the log-rank and the Breslow (generalized Wilcoxon) tests. Multivariate Cox backward stepwise regression analyzes were performed including all of the variables identified as significant on univariate Cox regression analyzes. Hazard ratios (HRs) and their 95% confidence intervals (CIs) were calculated. *p* values < 0.05 were considered statistically significant. Data analyzes were performed by applying Statistical Package for the Social Science (SPSS, version 25, IBM SPSS Statistics, Chicago, IL, United States) and GraphPad (version 6, GraphPad Software, San Diego, CA, United States) statistical software.

## Results

### Clinical Features

Overall, in our cohort of 112 patients there were 58 males (51.8%) and 54 females (48.2%), with a median age of 68 years (range 34–92 years). Seventeen patients (15.2%) had a cirrhotic liver, and 36 patients (32.1%) received adjuvant chemotherapy after resection/biopsy. Patients had a median follow-up of 1.7 years (range 0.3–8.8 years). Lymphadenectomy was performed in 33/90 patients who underwent surgery. Clinical and laboratory features of all the patients are summarized in Table 1. Age, sex, the presence of underlying chronic liver diseases, and the administration of adjuvant chemotherapy were not related to patients’ prognosis. Furthermore, overall and disease-free survivals were not different between patients with or without cirrhosis, probably because of the low number of cirrhotic patients in our cohort.

**TABLE 1:** Clinical and laboratory features of the patients.

Feature	*N* = 112
**Age [years]**	
Median (range)	68(34−92)
**Sex N (%)**	
*Males*	58 (51.8)
*Females*	54 (48.2)
**Ferritin [ng/ml]**	
Median (range)	260.5(53−943.5)
**Underlying diseases N (%)**	
*HBV hepatitis*	6 (5.4)
*HCV hepatitis*	11 (9.8)
*Alcoholic hepatitis*	6 (5.4)
*NAFLD/NASH*	6 (5.4)
*Cryptogenic cirrhosis*	1 (0.9)
**Cirrhosis N (%)**	17 (15.2)
**Adjuvant chemotherapy N (%)**	36 (32.1)
**Recurrence N (%)**	60 (53.6)
**Exitus N (%)**	73 (65.2)

*HBV, hepatitis B virus; HCV, hepatitis C virus; NAFLD, non-alcoholic fatty liver disease; NASH, non-alcoholic steatohepatitis.*

### Histological Features and Clinical-Pathological Correlations

Macroscopically, all cases were mass forming, while, histologically, there were 82 cases (73.2%) of small duct type ICC and 30 cases (26.8%) of large duct type ICC. We observed vascular invasion in 78 out of 112 patients (69.6%), while perineural invasion was present in 50/112 cases (44.6%), as reported in Table 2. Among the 33 patients who underwent lymphadenectomy, 15 had lymph node metastases. Forty-seven patients out of the 90 who underwent surgery (52.2%) had a complete surgical excision of the tumor (R0 cases), while the remaining 43 patients (47.8%) showed a microscopic neoplastic infiltration of the resection margin (R1 cases).

**TABLE 2:** Histological features of the patients.

Feature	*N* = 112
**Histotype**	
N (%)	
*Small duct type*	82 (73.2)
*Large duct type*	30 (26.8)
**Grade**	
N (%)	
*G1*	15 (13.4)
*G2*	49 (43.8)
*G3*	48 (42.8)
**T stage [*N* = 90]**	
N (%)	
*T1a*	13 (14.4)
*T1b*	11 (12.2)
*T2*	46 (51.1)
*T3*	13 (14.4)
*T4*	7 (7.9)
**Vascular invasion N (%)**	78 (69.6)
**Perineural invasion N (%)**	50 (44.6)
**Lymph node metastasis [*N* = 33] N (%)**	15 (45.5)
**Resection margin status [*N* = 90]**	
N (%)	
*R0*	47 (52.2)
*R1*	43 (47.8)

As expected, grading and the presence of perineural and vascular neoplastic invasion were all related to worse overall (*p* = 0.009, *p* < 0.0001, and *p* = 0.004, respectively) and disease-free survivals (*p* = 0.01, *p* = 0.002, and *p* = 0.001, respectively). An advanced T stage (i.e., T stage 3 and 4) was related to a reduced overall survival (*p* = 0.06). We failed to find any correlation between patients’ prognosis and histotype or resection margin status. We did not observe differences in survival times between cases with or without lymph node metastases.

### Immunohistochemical Features and Clinical-Pathological Correlations

In our cohort, most of the cases showed GPX4 expression, either 1 + (43/112; 38.4%) or 2 + (46/112; 41.1%), while only 23 cases (20.5%) were completely negative. Twenty-two patients out of 112 (19.6%) showed 1 + TFR1 expression, while 7 cases showed a 2 + TFR1 positivity (6.3%). Intratumoral iron deposits were observed in only 2 cases (1.8%); among them, one showed a focal and one a diffuse deposition. Interestingly, the case that showed diffuse iron deposits had both a strong TFR1 expression and a completely negative GPX4 stain (Supplementary Figure 1). Taken together, these findings suggest an inhibition of the ferroptotic cascade in ICC. Most of our cases showed either a 1 + (56/112; 50%) or 2 + (51/112; 45.5%) STAT3 expression, with only 5 cases (4.5%) showing a completely negative reaction, suggesting an activation of the inflammatory pathway in our ICC cohort. Histochemical and immunohistochemical expressions of the different markers are summarized in Table 3.

**TABLE 3:** Histochemical and immunohistochemical expression of the different markers in ICC cases.

Marker	*N* = 112
**GPX4**	
N (%)	
*0*	23 (20.5)
*1* +	43 (38.4)
*2* +	46 (41.1)
**TFR1**	
N (%)	
*0*	83 (74.1)
*1* +	22 (19.6)
*2* +	7 (6.3)
**Intratumoral iron deposits**	
N (%)	
*Absent*	110 (98.2)
*Present*	2 (1.8)
**STAT3**	
N (%)	
*0*	5 (4.5)
*1* +	56 (50)
*2* +	51 (45.5)

Statistical analyzes showed a direct correlation between GPX4 expression and the presence of poor prognostic histological parameters, that is grading (*p* < 0.0001) and perineural (*p* = 0.03) and vascular invasion (*p* < 0.0001). We also found a direct association between the presence of the same unfavorable histological factors and STAT3 expression (*p* = 0.001, *p* = 0.04, and *p* = 0.07 for grading, perineural invasion, and vascular invasion, respectively), confirming what previously reported (4, 10). No associations were found between the same histological features and TFR1 expression, neither between them and the presence of iron deposits. No correlations were found between marker expression (and iron deposits) and tumor T stage. We observed a direct association between STAT3 and GPX4 expression (*p* < 0.0001) (Supplementary Figure 2), suggesting inhibition of ferroptosis in ICCs with an inflammatory background. Accordingly, we found an inverse correlation between STAT3 and TFR1 expression (*p* = 0.04). We failed to find any correlation between TFR1 and GPX4 expression, as well as between ferroptosis markers and STAT3 expression and the presence of intratumoral iron deposits.

Age, sex, the presence of any underlying chronic liver disease, the presence of cirrhosis, ferritin levels, tumor histotype, and the presence of nodal metastases were not related to the expression of any of the markers, neither to the presence of iron deposits.

**FIGURE 4:**
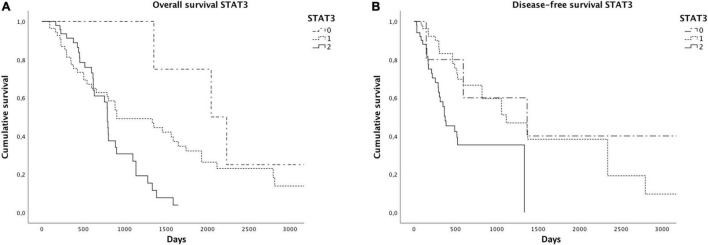
Kaplan–Meier curves showed reduced overall **(A)** and disease-free survivals **(B)** in cases with higher STAT3 expression.

**FIGURE 5:**
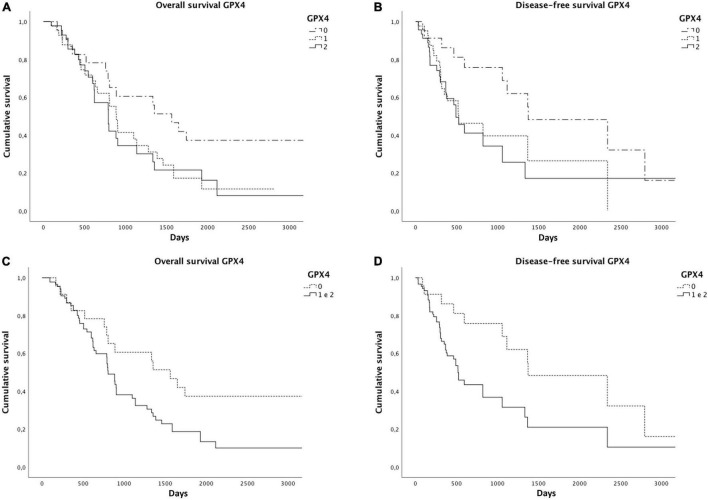
Significantly reduced overall **(A)** and disease-free survivals **(B)** were observed in cases with higher GPX4 expression. Considering GPX4 1 + and 2 + positive cases together, the association becomes even stronger for both overall **(C)** and disease-free survivals **(D)**, as shown by the Kaplan–Meier curves.

As already reported in the literature, STAT3 expression was related to a worse overall (*p* = 0.02) and disease-free survival (*p* = 0.001) (Figure 4). We found significantly reduced overall (*p* = 0.06) and disease-free survivals (*p* = 0.04) in cases with a higher GPX4 expression, as shown by the Kaplan–Meyer curves (Figure 5). Considering GPX4 1 + and 2 + positive cases together, the association becomes even stronger for both overall and disease-free survival (*p* = 0.03 and *p* = 0.01, respectively) (Figure 5), suggesting that mild GPX4 presence is enough to inhibit ferroptosis and reduce survival times. TFR1 expression and the presence of iron deposits were not related to survival times.

### Molecular Features and Clinical-Pathological Correlations

Molecular analysis results are reported in Table 4. As shown, they revealed *IDH1* point mutations in 15 out of 76 tested cases (19.7%), of which 14 involving codon 132 and one involving codon 105. Only one case showed a point mutation in *IDH2* codon 172 (1.3%). In 9 cases (11.8%) molecular tests described a single nucleotide polymorphism (SNP) in codon 105 of the *IDH1* gene, causing a change in the nucleotide sequence from GGC [Gly] to GGT [Gly] (C > T). Among them, two cases showed both a point mutation in *IDH1* (codon 132) and the *IDH1*^105*GGT*^ SNP. No relationships were found between the presence of IDH1 point mutations and the presence of the *IDH1*^105*GGT*^ SNP.

**TABLE 4:** *IDH1* and *IDH2* gene status in ICC cases.

Adequacy	*N* = 90
Inadequate samples	14/90 (15.6%)
Adequate samples	76/90 (84.4%)
**Molecular alterations**	***N* = 76**
*IDH1* point mutations*	15/76 (19.7%)
*Arg132Cys*	6/76 (7.9%)
*Arg132His*	2/76 (2.6%)
*Arg132Val*	1/76 (1.3%)
*Arg132Ser*	1/76 (1.3%)
*Arg132X*	4/76 (5.2%)
*Gly105Asn*	1/76 (1.3%)
*IDH1*^105*GGT*^ SNP*	9/76 (11.8%)
***IDH2* Arg172X**	1/76 (1.3%)
***IDH1-2* WT**	53/76 (69.7%)

*2 cases showed both an IDH1 point mutation in codon 132 (Arg132Cys and Arg132His) and IDH1^105GGT^ SNP. SNP, single nucleotide polymorphism; WT, wild type.

Statistical analyzes showed an inverse correlation between the presence of *IDH1*^105*GGT*^ SNP and tumor grading (*p* < 0.0001). On the contrary, a direct relationship was found between tumor grading and the wild type status of *IDH1-2* genes (*p* = 0.004). No associations between tumor grading and the presence of point mutations in *IDH1-2* genes were observed. We also failed to find any correlation between *IDH1-2* status and age, sex, the presence of any underlying chronic liver disease, the presence of cirrhosis, ferritin levels, tumor histotype, the presence of perineural and vascular invasion, T stage and the presence of nodal metastases.

Furthermore, GPX4 expression was inversely correlated to the presence of *IDH1*^105*GGT*^ SNP (*p* = 0.001) (Supplementary Figure 3), while it was directly related to the presence of an *IDH1-2* point mutation (*p* = 0.06) or wild type *IDH1-2* (*p* = 0.04). No associations were found between *IDH1-2* status and STAT3 and TFR1 expression, neither between *IDH1-2* status and intratumoral iron deposits.

**FIGURE 6:**
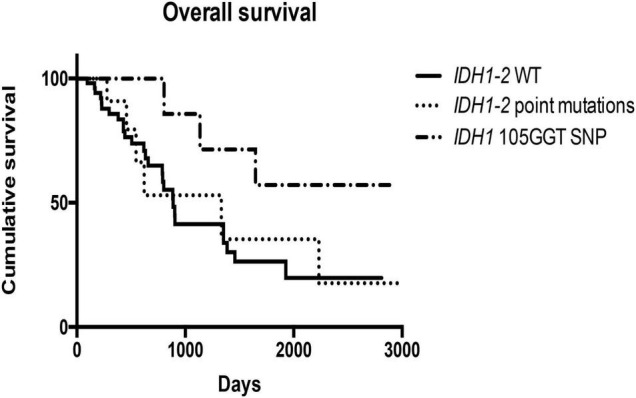
Kaplan–Meier curves showed different overall survivals in cases bearing *IDH1*^105*GGT*^ SNP, *IDH1-2* point mutations and wild type *IDH1-2*.

Finally, cases with *IDH1*^105*GGT*^ SNP showed a better overall survival than cases with wild type *IDH1-2* (1,648 days vs. 887 days; *p* = 0.04) and cases with *IDH1-2* point mutations (1,648 days vs. 1,333 days; *p* = 0.09) (Figure 6). We did not find any relationship between *IDH1-2* status and disease-free survival times. The multivariate Cox regression analysis, including variables that were identified as significant on univariate survival analyzes (grading, perineural and vascular neoplastic invasion, T stage, STAT3 and GPX4 expression, and *IDH1*^105*GGT*^ SNP) showed that only the presence of perineural invasion is an independent predictor of a worse overall survival [*p* < 0.0001, HR = 3.64 (95% CI: 1.86–7.11)], while the multivariate Cox regression analysis including grading, perineural and vascular neoplastic invasion, and STAT3 and GPX4 expression found that STAT3 expression and vascular invasion are independent predictors of a reduced disease-free survival [*p* = 0.03, HR = 1.81 (95% CI: 1.05–3.14), and *p* = 0.05, HR = 1.97 (95% CI: 1.02–3.84), respectively] (Supplementary Table 1).

## Discussion

In this study, we describe for the first time that in most inflammatory ICCs ferroptosis seems to be not active, and that its activation may depend on *IDH1-2* status. In our cohort, GPX4 was overexpressed in almost 80% of the cases, suggesting an inhibition of the ferroptotic cascade in most of our patients. GPX4 expression was found to be related to the presence of histological poor prognostic features, namely high grade and vascular and perineural invasion, and to reduced overall and disease-free survivals, implying that ferroptosis inhibition confers a worse prognosis, as expected.

We also observed that STAT3 was overexpressed in more than 90% of cases, indicating that they can be classified as inflammation-related ICC, according to the molecular classification proposed by Sia et al. (9). We confirm the poor prognostic role of STAT3 already reported in the literature (4, 10). In fact, we found a correlation between its expression and the presence of histological poor prognostic features, and worse overall and disease-free survivals in cases with higher STAT3 expression. Since this subclass of ICC is characterized by a pro-inflammatory and anti-apoptotic molecular milieu, and that pro-apoptotic drugs demonstrated only mild improvements in patients’ prognosis and a frequent development of chemoresistance (4, 7, 11), the induction of other different cell death types, including ferroptosis, is a possible alternative way to kill resistant tumor cells. In line with this hypothesis, the high GPX4 levels found in our patients suggest that ferroptosis could be pharmacologically induced, by acting directly on GPX4 inhibition.

Molecular analyses on our cohort demonstrated *IDH1* (codon 132 or 105) or *IDH2* (codon 172) point mutations in nearly 20% of the cases, in line with the literature (3, 4, 14–17). Surprisingly, we found a SNP on codon 105 of the *IDH1* gene in 12% of our cases, which was never reported in ICC before. Patients bearing this molecular feature had a good histological profile, namely low histological tumor grade, and longer overall survival times. Synonymous SNPs are point mutations that cause a nucleotide change, which do not alter the amino acid sequence of the protein. However, sometimes they may lead to a protein defect and have functional consequences (24). The *IDH1*^105*GGT*^ SNP we described in our cohort was previously reported in acute myeloid leukemia, gliomas, and thyroid tumors, and have a poorly understood role in tumorigenesis (24–27). Indeed, it was linked to an adverse prognosis in acute myeloid leukemia and glioblastomas, while it seemed to confer longer survivals in patients with grade II or III gliomas (28, 29), in line with our results. The explanation of this finding is not clear, since the biologic consequences of this SNP remain speculative. It has been hypothesized that *IDH1*^105*GGT*^ SNP may alter *IDH1* mRNA stability or increase mRNA levels, leading to altered NADPH production (24, 28, 30), but further studies are needed to elucidate this issue. In line with previously reports (24, 28, 30), in two of our cases we described the concomitant presence of the *IDH1*^105*GGT*^ SNP and a point mutation in *IDH1* (codon 132). It is known that, even if *IDH1*^105*GGT*^ SNP is very close to codon 132, no correlation between the two molecular alterations exists (24, 30), as we confirm in our cohort.

The inverse correlation we observed between the presence of *IDH1*^105*GGT*^ SNP and GPX4 levels may suggest an activation of the ferroptotic cascade in ICCs bearing this molecular feature, and eventually explains the longer overall survivals observed in these patients. On the other side, we failed to confirm the association between *IDH1*^*R*132*C*^ mutation and reduced GPX4 levels reported by Wang et al. (21), but this might be explained by the different experimental conditions we worked under (cell lines versus ICC tissue and *in vitro* versus *in vivo*). Considering our survival data, it is possible that both *IDH1*^105*GGT*^ SNP and *IDH1*^*R*132*C*^ mutation act on GPX4 in different ways or with different intensity, since *IDH1*^*R*132*C*^ mutation-bearing cases showed overall survival times shorter than those seen in *IDH1*^105*GGT*^ SNP cases, but longer than those of patients with wild type *IDH1-2*. However, molecular mechanisms explaining how *IDH1*^105*GGT*^ SNP act on GPX4 level reduction are unknown and impossible to infer basing on our data. So additional *in vitro* studies are indispensable to address this key issue. Moreover, the number of patients overall bearing the *IDH1*^105*GGT*^ SNP in our cohort is low, and this limits the strength of our data, so additional studies based on larger cohorts may be of help to confirm our results. It is of interest to note that patients bearing *IDH1*^105*GGT*^ SNP are not expected to respond to GPX4-inhibitor drugs, such as RSL3, since GPX4 levels are already very low in these cases. However, as Wang et al. reported on *IDH1*^*R*132*C*^-mutated cell lines (21), *IDH1*^105*GGT*^ SNP-bearing ICCs may respond to ferroptosis-inducers acting on GSH levels, such as erastin. Therefore, knowing the molecular background in ICC patients is fundamental to choose the appropriate pharmacological therapy to induce tumor cell death, particularly in cases developing drug-resistance.

In conclusion, our study demonstrates for the first time that in most inflammatory ICCs ferroptosis seems to be not active, and that its triggering may be related to some molecular features of the tumor. *IDH1-2* status is essential to determine whether (and which type of) ferroptosis-inducer drugs might be useful in ICC patient treatment, especially in drug-resistant cases.

## Data Availability Statement

The original contributions presented in this study are included in the article/Supplementary Material, further inquiries can be directed to the corresponding author.

## Ethics Statement

The studies involving human participants were reviewed and approved by Ethics Committee for Clinical Research—University Hospital of Padova, Italy. Written informed consent for participation was not required for this study in accordance with the national legislation and the institutional requirements.

## Author Contributions

SS contributed to conception and design, acquisition of data, analysis and interpretation of data, writing and revision of the manuscript. DS contributed to conception and design, acquisition of data, analysis and interpretation of data, and revision of the manuscript. LF, GZ, and EG contributed to revision of the manuscript. MN contributed to development of methodology and technical and material support. GG contributed to development of methodology, analysis and interpretation of data, and technical and material support. MG contributed to conception and design, interpretation of data, revision of the manuscript, and study supervision. All authors contributed to the article and approved the submitted version.

## Conflict of Interest

The authors declare that the research was conducted in the absence of any commercial or financial relationships that could be construed as a potential conflict of interest.

## Publisher’s Note

All claims expressed in this article are solely those of the authors and do not necessarily represent those of their affiliated organizations, or those of the publisher, the editors and the reviewers. Any product that may be evaluated in this article, or claim that may be made by its manufacturer, is not guaranteed or endorsed by the publisher.
