# Supplementary figures and images for: Ferroptosis in Intrahepatic Cholangiocarcinoma: IDH1105GGT Single Nucleotide Polymorphism Is Associated With Its Activation and Better Prognosis
Source: Front Med (Lausanne). 2022 Jul 8;9:886229. doi: 10.3389/fmed.2022.886229 (PMC9304620; doi:10.3389/fmed.2022.886229)

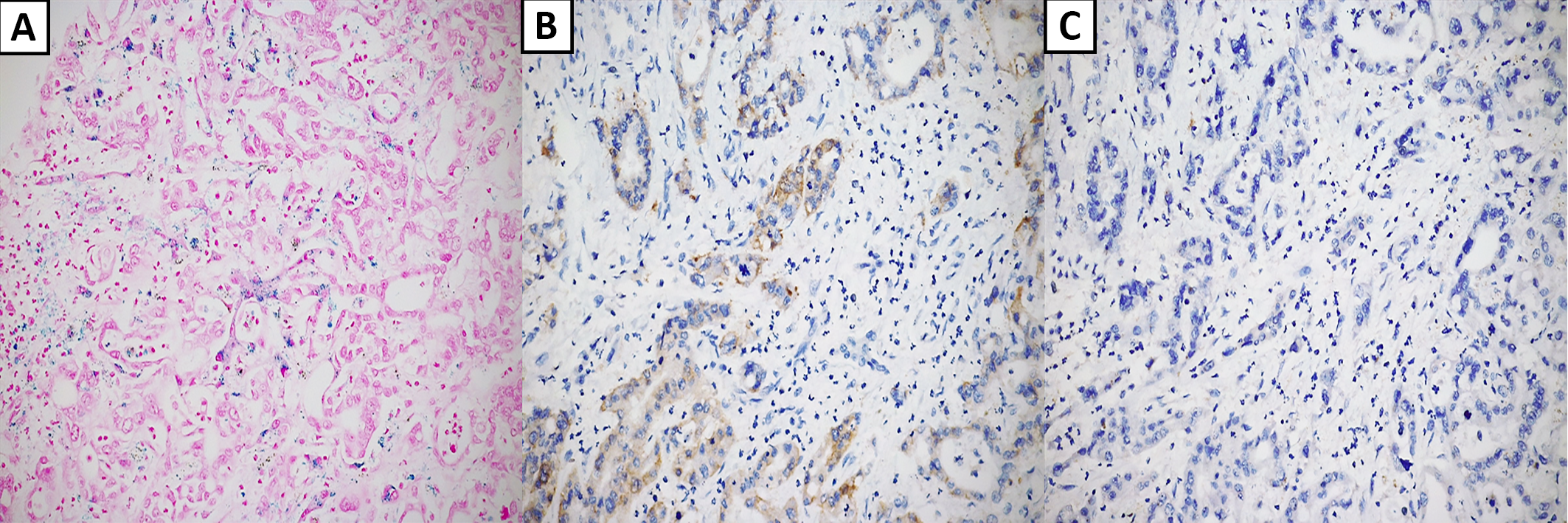

Supplement: Supplementary file 1 [file Image_1.tif]

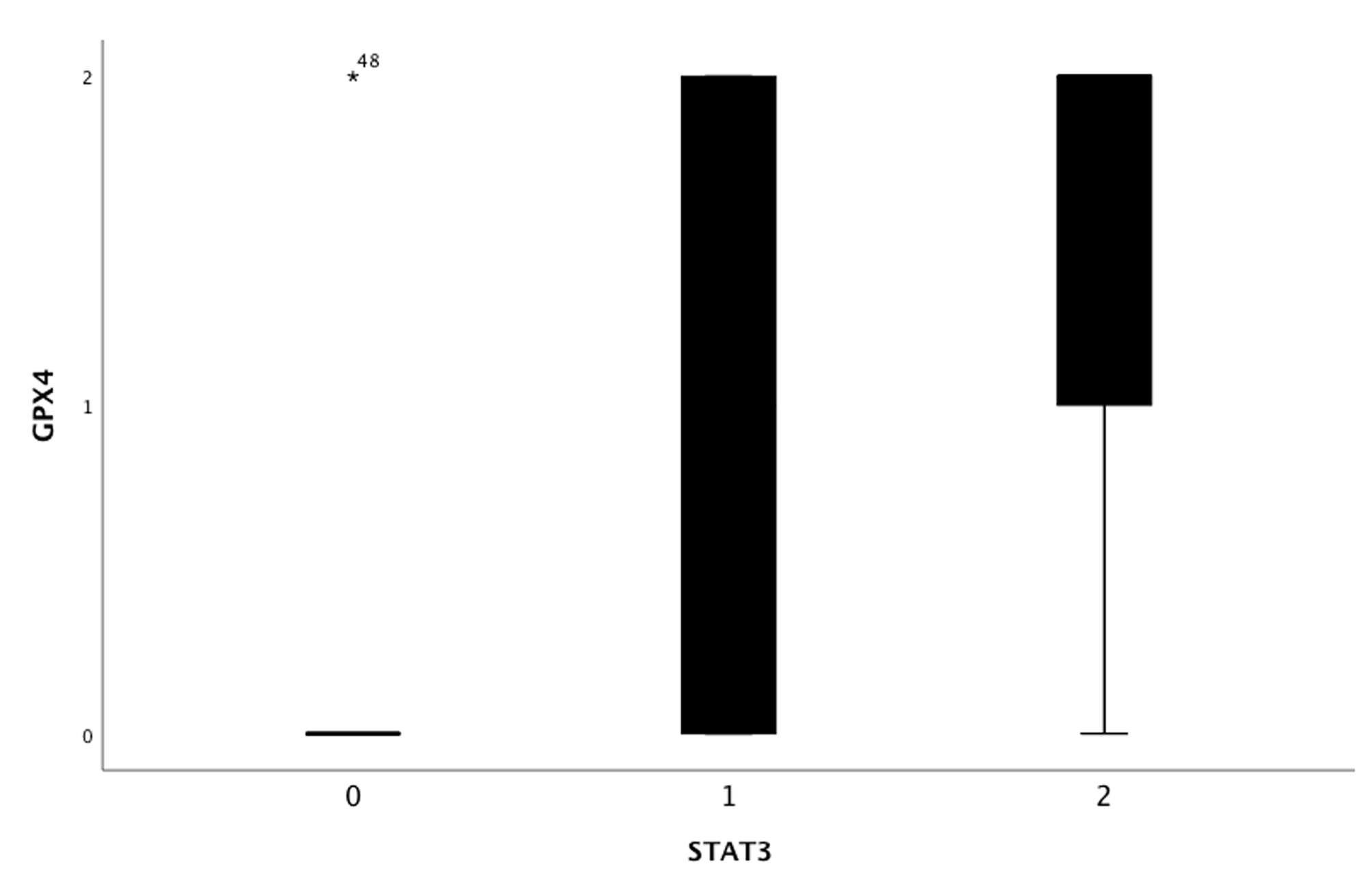

Supplement: Supplementary file 2 [file Image_2.tif]

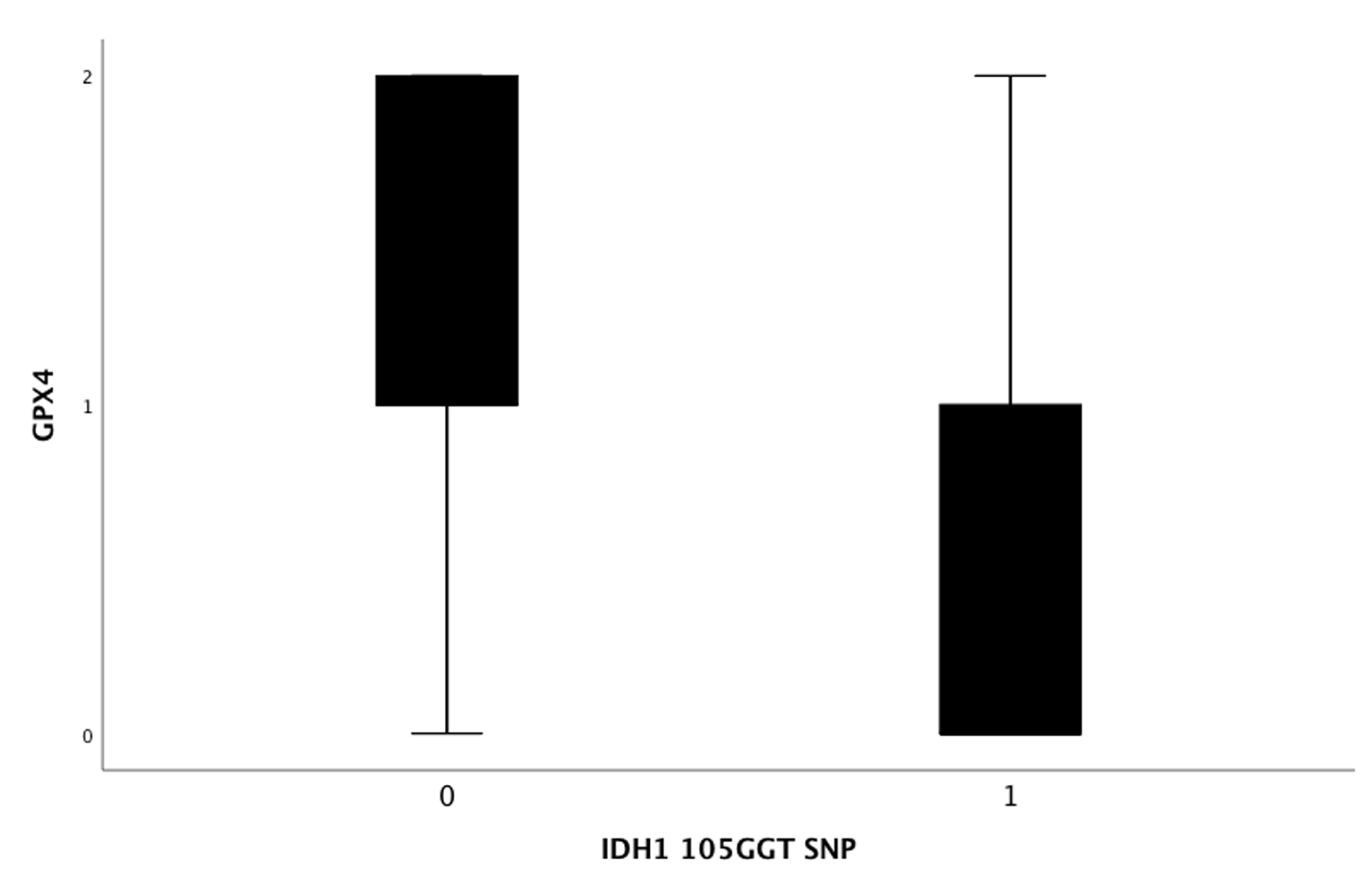

Supplement: Supplementary file 3 [file Image_3.tif]
