# Supplementary material for: Ferroptosis in Intrahepatic Cholangiocarcinoma: IDH1105GGT Single Nucleotide Polymorphism Is Associated With Its Activation and Better Prognosis
Source: Front Med (Lausanne). 2022 Jul 8;9:886229. doi: 10.3389/fmed.2022.886229 (PMC9304620; doi:10.3389/fmed.2022.886229)
Supplement: Supplementary file 4 [file Table_1.DOCX]

Supplementary Material

# Supplementary Figure Legends

**Supplementary Fig. 1.** One ICC case showed diffuse intratumoral iron deposits (A; original magnification 20x), a strong TFR1 expression (B; original magnification 20x), and a completely negative GPX4 stain (C; original magnification 20x), suggesting the activation of the ferroptotic cascade.

**Supplementary Fig. 2.** The box plot represents the direct correlation between STAT3 and GPX4 expression (p <0.0001).

**Supplementary Fig. 3.** GPX4 expression was inversely associated to the presence of *IDH1*^105GGT^ SNP (p = 0.001), as shown in the box plot.

**Supplementary Table 1.** Multivariate analyses results.

|  | **Overall survival** | | **Disease-free survival** | |
| --- | --- | --- | --- | --- |
| **Variables** | **HR (95% CI)** | ***p*** | **HR (95% CI)** | ***p*** |
| *Grading* | 1.32 (0.81-2.14) | n.s. | 1.37 (0.89-2.1) | n.s. |
| *Perineural invasion* | 3.64 (1.86-7.11) | <0.0001 | 1.68 (0.91-3.11) | n.s. |
| *Vascular invasion* | 1.28 (0.58-2.83) | n.s. | 1.97 (1.02-3.84) | 0.05 |
| *T stage* | 1.19 (0.89-1.59) | n.s. | / | / |
| *STAT3* | 1.27 (0.69-2.34) | n.s. | 1.81 (1.05-3.14) | 0.03 |
| *GPX4* | 1.06 (0.51-2.22) | n.s. | 1.05 (0.43-2.56) | n.s. |
| *IDH1^105GGT^ SNP* | 0.5 (0.2-1.24) | n.s. | / | / |
